# Genetic and Dietary Influences on Metabolic Traits in Gilthead Seabream (*Sparus aurata*)

**DOI:** 10.3390/genes17050550

**Published:** 2026-05-05

**Authors:** Stavroula Oikonomou, Rafael Angelakopoulos, Maria Tekeoglou, Andreas Tsipourlianos, Zoi Kazlari, Dimitrios Loukovitis, Arkadios Dimitroglou, Themistoklis Giannoulis, Zissis Mamuris, Dimitrios Chatziplis, Katerina A. Moutou

**Affiliations:** 1 Laboratory of Agrobiotechnology and Inspection of Agricultural Products, Department of Agriculture, International Hellenic University, Alexander Campus, Sindos, P.O. Box 141, 57400 Thessaloniki, Greece; valiaekonomou@hotmail.com (S.O.); mariatek55@gmail.com (M.T.); zoikaz@hotmail.gr (Z.K.); 2 Research Institute of Animal Science, ELGO Demeter, Paralimni, 58100 Giannitsa, Greece; 3 Faculty of Health, Medicine and Life Sciences, Department of Pharmacology and Toxicology, School for Nutrition and Translational Research in Metabolism (NUTRIM), Maastricht University, 6229 ER Maastricht, The Netherlands; 4 Laboratory of Genetics, Comparative and Evolutionary Biology, Department of Biochemistry and Biotechnology, University of Thessaly, Biopolis, 41500 Larissa, Greece; rangelak@uth.gr (R.A.); zmamur@uth.gr (Z.M.); kmoutou@bio.uth.gr (K.A.M.); 5 Perrotis College, American Farm School, Marinou Antipa 54, Thermi, P.O. Box 60097, 57001 Thessaloniki, Greece; 6 Department of Fisheries and Aquaculture, School of Agricultural Sciences, University of Patras, New Buildings, PC, 30200 Messolongi, Greece; dloukovi@upatras.gr; 7 Laboratory of Applied Hydrobiology, Department of Animal Science, Agricultural University of Athens, Iera Odos 75, 11855 Athens, Greece; a.dimitroglou@aua.gr; 8 Laboratory of Biology, Genetics and Bioinformatics, Department of Animal Science, University of Thessaly, Greece Gaiopolis, 41334 Larissa, Greece; thgianno@uth.gr

**Keywords:** gilthead seabream, genetic parameters, plant-based diet, metabolic traits, hepatic gene expression, SNP markers, aquaculture nutrition

## Abstract

Background/Objectives: In gilthead seabream, the transition from fish meal/oil-based diets to diets with partial plant-based replacement is gaining ground due to price fluctuations and environmental concerns. Most studies focus on the dietary effects on important commercial traits such as body weight and fat deposition, while metabolic traits and their underlying genetic and transcriptional regulation remain largely unexplored. Methods: In the present study, the response of metabolic traits (protein, cholesterol, and triglycerides levels) was measured in gilthead seabream of different genetic backgrounds at 15 (D15) and 30 days (D30) after a shift from a fish meal/oil-based diet (FM) to a plant-based (PP) diet. Results: Moderate heritability of total protein and triglyceride content of blood was estimated on D30. Significantly positive genetic correlations were observed between triglyceride D30 content and final weight and muscle fat. No significant genotype-by-diet interaction effects were detected. At the end of the production cycle, final body weight and fat were recorded, and hepatic expressions of *ghri*, *ghrii*, *igf1* and *ttr* genes were measured in a subpopulation of 160 fish. An overall negative correlation was recorded between the hepatic expression of *igf1* and final weight, whereas strong positive correlations were observed between the expression of all hepatic genes measured. In the same population, fourteen SNPs located in the 3′ UTR of *ghrii* and *igf1* genes were genotyped and analyzed in two ways, as a sum-of-risk score and individually as predictors for body weight, muscle fat, metabolic traits and hepatic expression levels. The sum-of-risk score was significantly associated with muscle fat and *ttr* expression. Studying the effect of each SNP independently, two SNPs in the *igf1* gene were associated with *ghrii* expression levels and one SNP in *igf1* gene was associated with triglyceride levels at day 15 (Trigl_D15) while one SNP in *ghrii* was associated with *ttr* expression levels. Focusing on the diet, it was significantly associated with final weight, muscle fat, protein (D30) and triglycerides levels, and hepatic expression levels of *ghrii*.

## 1. Introduction

Fish meal and fish oil are among the most important ingredients in aquafeeds due to their high nutritional value and balanced amino acid and fatty acid profiles. However, their availability is limited, and their cost fluctuates considerably, posing economic and sustainability challenges for aquaculture production. To reduce dependence on these marine-derived feed resources, extensive research has focused on the partial or complete replacement of fish meal and fish oil with plant-based ingredients [1]. In gilthead seabream (*Sparus aurata*), complete replacement often results in impaired growth performance and metabolic disturbances, whereas partial replacement strategies generally lead to more favorable outcomes with reduced negative effects on growth and feed utilization [2,3,4].

Beyond feed formulation, fish performance is strongly influenced by their genetic background and many commercial aquaculture operations implement selective breeding programs to improve the efficiency of economically important traits such as body weight and growth rate. In Mediterranean aquaculture, genetics research aiming at estimating the heritability of traits of commercial interest has focused on European seabass (*Dicentrarchus labrax*) [5,6,7,8], meagre (*Argyrosomus regius*) [9,10,11], and gilthead seabream [12,13,14,15,16,17]. The interaction between genetic background and feed composition remains a critical challenge for breeding programs and it is gaining importance rapidly for commercial operations [18].

In gilthead seabream, the transition from fish meal/fish oil-based diets to diets with partial plant-based replacement can substantially affect the performance ranking of individuals and families within commercial populations. This issue was explicitly addressed by Oikonomou et al. [17], who investigated the genotype × diet (G × D) interactions in a large commercial population and demonstrated significant re-ranking of selection candidates (broodstock candidates) when different diets were used. These findings highlighted that individuals respond differently to dietary composition depending on their genetic background. However, that study focused exclusively on growth-related traits, leaving other physiologically relevant traits unexplored.

Blood circulating levels of triglycerides, cholesterol, and total protein constitute key markers of energy allocation, lipid metabolism, nutritional status, and overall physiological condition in fish [19]. Such markers have been linked to growth performance, body composition, and robustness in several teleost species, as they reflect the balance among nutrient intake, storage, and utilization and they are responsive to nutritional changes [20,21,22,23,24]. Most importantly, such markers can be measured using minimally invasive blood sampling, making them attractive candidates as surrogate markers for performance and health in selective breeding programs [25]. Despite their potential relevance, there is currently limited information on the heritability of these indicators in gilthead seabream, their genetic correlations with body weight and muscle fat, and their response to contrasting dietary regimes.

Feed composition is known to influence metabolic regulation primarily through the liver, a central organ in controlling lipid metabolism, energy homeostasis, and endocrine signaling [26]. In fish, growth and metabolic adaptation to nutrition are largely mediated by the growth hormone–insulin-like growth factor (GH–IGF) axis, which integrates nutritional cues with somatic growth and nutrient partitioning [27]. As a result, the somatotropic axis has been used as an endocrine marker of the effectiveness of alternative feed formulations [26,27]. Key components of this axis include insulin-like growth factor 1 (*igf1*) and the growth hormone receptors (*ghri*, *ghrii*), which play central roles in regulating growth and metabolic responses to dietary changes in the liver and white muscle [27]. Fast growth has been the top targeted trait in breeding selection programs, often accompanied by high fat content [28]. However, there is evidence from rainbow trout that selecting for lean body mass, i.e., for fast weight gain and against muscle lipid percentage, can be as effective as selecting for efficient conversion of ingested protein into protein weight gain [29]. Transthyretin (*ttr*) is the sole plasma protein closely associated with lean body mass in humans and a clinical marker of nutritional status [30]. TTR is a carrier protein of thyroid hormone and retinol-binding protein 4, and in gilthead seabream, it is primarily produced in the liver [31]. Evidence from fasting–refeeding trials in gilthead seabream shows that ttr transcript levels respond to dietary changes along with circulating thyroid hormone levels [32].

The first objective of this study was to estimate genetic parameters (heritability and genetic correlations) for early circulating metabolic traits (triglycerides, cholesterol, and total protein) in gilthead seabream and to assess the presence of genotype × diet interactions under diets differing in fish meal and fish oil inclusion. This analysis was conducted using 584 individuals originating from a large commercial breeding population reared using two diets in a split-family design as previously described by Oikonomou et al. [17].

The second objective was to investigate the effect of genetic variation in key endocrine genes on metabolic regulation and performance. Variation in gene regulatory regions, rather than coding sequence alone, is increasingly recognized as an important source of phenotypic diversity [33,34]. However, the extent to which regulatory variation in key endocrine genes contributes to metabolic traits, gene expression, and growth performance under different dietary conditions in gilthead seabream remains largely unexplored. In this direction, we examined whether SNPs located in the 3′ UTRs of *igf1* and *ghrii* are associated with (i) hepatic expression levels of *igf1*, *ghri*, *ghrii*, and *ttr*, (ii) circulating blood metabolic traits, and (iii) growth performance and muscle fat content. This analysis was performed in a subset of 160 individuals from the same population. By integrating quantitative genetic analyses with hepatic gene expression and regulatory genetic variation, this study aims to provide a possible comprehensive framework linking diet, metabolism, endocrine regulation, and genetic background in gilthead seabream.

## 2. Materials and Methods

### 2.1. Ethical Statement

All examined biological materials derived from fish reared and harvested at commercial farms are registered for aquaculture production in EU countries. Animal sampling followed routine procedures and the samples were collected by qualified staff members from standard production cycles. The legislation and measures implemented by the commercial producers complied with existing national and EU (Directive 1998/58/EC) legislation (protection of animals kept for farming).

### 2.2. Fish Population and Feed

In July 2018, a total of 4250 fish of 118 families in the company’s commercial breeding program were randomly distributed into sea cages in a split-family experiment and assigned into two groups (cages): the FM group was continuously fed on commercial diet (FM), whereas the PP group was subjected to a dietary shift from the FM to the PP from 225 days post-hatching (DPH) to the end of the rearing period (549 DPH). Consequently, the effect of diet was confounded with the effect of cage and thus, only one fixed effect was used in the data analysis.

The FM was formulated using marine raw materials, including 24.6% standard fish meal, 4.6% fish oil, and 6.5% salmon oil. In contrast, the PP contained 8.1% fish meal and 5% fish oil, with higher inclusions of plant-based raw materials (for further details, see [17,20,21]. The experimental duration was more than 18 months, and seawater temperature fluctuated from 15.2 to 28.0 ◦C. At 549 DPH, the average weight was 650.20 g (±118.71) and muscle fat content was 16.28% (±4.47) [17].

In the present study, a sub-sample of 584 fish from 20 families (11 full sib and 9 half sib families, approximately 30 offspring per family, equally distributed across the cages and dietary groups) was selected for the present analysis. The 20 families were selectively bred from individuals displaying extreme phenotypes. Ten families originated from progeny with high weight gain and low within-family variance in weight (CV: 10–12%), whereas the remaining ten families came from progeny with low weight gain and high within-family variance (CV: 25–26%).

### 2.3. Studied Phenotypes

The final body weight and the muscle fat content were measured at 549 DPH. The muscle fat content was recorded using a Distell fat meter (Distell FFM-692, Old Levenseat, Scotland, UK), by measuring four standard points on the same side for all fish and was expressed as the percentage of the body weight (FAT %). Fifteen days (D15) and thirty days (D30) after the dietary shift (change from FM to PP), a blood sampling was performed, and serum was collected from all 584 fish. Serum protein content, cholesterol, and triglyceride levels were measured using a colorimetric assay kit (Biosis, Athens, Greece cat. no: 000244) following the manufacturer’s protocol, with minor modifications [21]. Descriptive statistics of all these phenotype data were calculated in R. A list of all the studied phenotypes is shown in Table 1.

### 2.4. Estimation of Genetic Parameters

The heritability of all the metabolic factors, muscle fat content and body weight, along with their genetic/phenotypic correlations, was estimated using the Restricted Estimation of Maximum Likelihood method (REML). The analyses were performed using AIREMLF90 [35]. For the heritability estimation, a univariate animal model was applied to each phenotype (Model 1). To estimate genetic/phenotypic correlations, bivariate animal models were used to fit a different pair of phenotypes each time (Model 2).

The equation for Model 1 is:

***Υ* = *Χβ* + *Zu* + *e***

where ***Y*** corresponds to the vector of measurements for each trait, ***X*** is the incidence matrix relating records and fixed effect, ***β*** corresponds to the vector of the fixed effect (diet, 2 levels), ***Z*** is the incidence matrix relating records and random effects, ***u*** is the additive genetic effect utilizing the Pedigree Relationship Matrix (PRM) and it is illustrated as ~*N*(0, *Pσ_a_*^2^) (*P* is the PRM and *σ_a_*^2^ is the additive variance), and ***e*** is the residual.

The equation of Model 2 is:

$$\left[\begin{array}{c} y_{1}\\ y_{2} \end{array}\right]=\left[\begin{array}{cc} X_{1} & 0\\ 0 & X_{2} \end{array}\right]\left[\begin{array}{c} b_{1}\\ b_{2} \end{array}\right]+\left[\begin{array}{cc} Z_{1} & 0\\ 0 & Z_{2} \end{array}\right]\left[\begin{array}{c} u_{1}\\ u_{2} \end{array}\right]+\left[\begin{array}{c} e_{1}\\ e_{2} \end{array}\right]$$

where ***y*_1_** and ***y*_2_** are the vectors of measurements for each traits 1 and 2, respectively, ***X*_1_** and ***X*_2_** are incidence matrices relating records and fixed effects, ***b*_1_** and ***b*_2_** are fixed quantities including the underlying means for each trait, ***Z*_1_** and ***Z*_2_** are incidence matrices relating records and random effects, ***u*_1_** and ***u*_2_** are vectors of the additive genetic effects for traits 1 and 2, and finally, ***e*_1_** and ***e*_2_** are vectors of random errors. It was assumed that the random effects had zero means:

$$E\left[\begin{array}{c} y_{1}\\ y_{2} \end{array}\right]=\left[\begin{array}{cc} X_{1} & 0\\ 0 & X_{2} \end{array}\right]\left[\begin{array}{c} b_{1}\\ b_{2} \end{array}\right]$$

The variance structure of the model was assumed to be

$$var\left(\left[\begin{array}{c} u_{1}\\ u_{2}\\ e_{1}\\ e_{2} \end{array}\right]\right)=\left[\begin{array}{cccc} A\sigma_{a_{1}}^{2} & A\sigma_{\alpha_{12}} & 0 & 0\\ A\sigma_{a_{12}} & A\sigma_{a_{2}}^{2} & 0 & 0\\ 0 & 0 & I\sigma_{e_{1}}^{2} & I\sigma_{e_{12}}\\ 0 & 0 & I\sigma_{e_{12}} & I\sigma_{e_{2}}^{2} \end{array}\right]$$

where A is the numerator relationship matrix derived from the pedigree and I is an identity matrix, $\sigma_{a_{1}}^{2}$ and $\sigma_{a_{2}}^{2}$, are the additive genetic variances for traits 1 and 2, $\sigma_{a_{12}}$, is the additive genetic covariance between the two traits, $\sigma_{e_{1}}^{2}$, $\sigma_{e_{2}}^{2}$, are the residual variances, and $\sigma_{e_{12}}$ is residual covariance between the traits. In addition, covariances between additive genetic and residual effects were assumed to be zero [36].

### 2.5. Investigation of Genotype by Diet Interaction

A bivariate animal model was used to investigate genotype by diet (G × D) interactions for each possible pair of metabolic traits. For each metabolic trait, the phenotypes of fish fed the PP were treated as one trait, while the phenotypes of fish fed the standard commercial diet were considered as another [17]. In this analysis, Model 2 was used.

### 2.6. Liver Transcriptomic Analysis

At the end of the rearing period (549 DPH), 160 fish from both feed groups were selected and sacrificed, and liver tissue was extracted and stored in RNAlater (hermofisher, Waltham, MA, USA). Out of the 20 families, fish from 10 families were selected based on their comparative Specific Growth Rate (SGR) patterns when fed the two feeds. Briefly, three SGR patterns were observed; in pattern A, FM and PP resulted in different SGR the period from September 2018 to January 2019; in pattern B, the two diets resulted in SGR differences only between September and November 2018; in pattern C, no variation in SGR was observed between the two diets at any time within the rearing period. Both extreme phenotypes were represented. Additional details on the zootechnical parameters of all 20 families are available on [21].

Total RNA was extracted using the E.Z.N.A.^®^ Total RNA Kit I (OMEGA bio-tek, Omega Bio-tek, Inc., Atlanta, GA, USA) according to the manufacturer’s protocol, followed by treatment with the DNAfree DNA Removal Kit (Thermo Scientific, Waltham, MA, USA) to eliminate any residual DNA. RNA quantity and quality were assessed using the Qubit™ RNA BR Assay Kit (Invitrogen, Carlsbad, CA, USA). cDNA synthesis was performed using the High-Capacity cDNA Reverse Transcription Kit with RNase Inhibitor (Thermo Scientific, Waltham, MA, USA), using 1 μg of DNase-treated total RNA and a combination of oligo(dT) and random primers. cDNA samples were diluted and stored at −80 °C until further use.

Gene expression levels of key metabolic genes, specifically *ghri*, *ghrii*, *igf1*, and *ttr*, were quantified in the liver using real-time PCR (Table 2). These genes were selected for their essential roles in metabolic regulation and growth. Genes *ghri* and *ghrii* encode growth hormone receptors that mediate the effects of growth hormone on metabolism and tissue development, while *igf1* (insulin-like growth factor 1) acts as a crucial downstream effector, regulating cell proliferation, differentiation, and protein synthesis. *ttr* (transthyretin) plays a key role in energy homeostasis by transporting thyroid hormones and retinol. Primers were designed using Primer3 (v.0.4.0) and Beacon Designer software (Table 2). PCR efficiency was assessed using a standard curve [37].

Real-time PCR was conducted in duplicate on a StepOne Plus PCR System (Thermo Scientific). Reactions were performed using KAPA SYBR FAST qPCR Master Mix (KAPA Biosystems, Wilmington, MA, USA, KK4602). The following cycling conditions were applied: an initial denaturation step at 95 °C for 5 min, followed by 40 cycles of amplification (20 s at 95 °C, 20 s at 60 °C), and a final melting curve step (15 s at 95 °C, 1 min at 50 °C, 15 s at 95 °C) to confirm the specificity of the reaction. A set of housekeeping genes (*ef1α*, *rpl13a*, *rps18*) was validated for stability using the RefFinder platform (https://www.ciidirsinaloa.com.mx/RefFinder-master/?type=reference, accessed on 11 March 2025).

The relative expression levels were calculated from the obtained Ct values using the formula *R*0 = *Threshold*/(1 + *Efficiency*) *Ct* and normalized using the geometric mean of the two most stable housekeeping genes [38]. All types of data failed the Shapiro–Wilk test for normality and were analyzed using non-parametric tests. Significant differences in gene expression between conditions were assessed using the Wilcoxon signed-rank test. Kendall’s rank correlation was performed to evaluate the statistical dependence between variables. All statistical analyses were conducted in R.

### 2.7. Genotyping and SNP Analysis of ghrii and igf1

SNP data was extracted from whole-genome sequencing (WGS) and RNA-seq generated in previous works in the laboratory of Genetics, Comparative and Evolutionary Biology (BioProjects ID: PRJNA1050571 and PRJNA1064006, respectively) and used to construct a VCF file [39]. To assess the potential impact of genetic variation on gene expression of *ghrii* and *igf1* genes, single-nucleotide polymorphisms located in the 3′ UTRs were extracted from this VCF file, as these regulatory regions can influence gene expression. Based on this criterion, 17 SNPs were selected for genotyping in individuals with corresponding gene expression data (Table 3). *igf1* and *ghrii* were prioritized for 3′ UTR SNP genotyping because their expression profiles showed the most promising trends in relation to diet and phenotype, while comparable patterns were not observed for *ghri* and *ttr*.

For the *igf1* gene, a custom pair of primers was used to amplify a fragment of its 3′ UTR region encompassing twelve (12) SNPs. Also, for the *ghrii* gene, a custom primer pair was used to amplify a 3′ UTR fragment containing five (5) SNPs. Primers used for genotyping are shown in Table 4. PCR amplifications were performed in 10 µL reaction volumes, each containing 8 µL of 2× Taq Master Mix (New England Biolabs, Ipswich, MA, USA), 0.5 µL of each primer (0.5 µM final concentration), and 1 µL of template DNA (~30 ng). The thermal cycling protocol was as follows: initial denaturation at 94 °C for 2 min; 30 cycles of 94 °C for 30 s, 62 °C for 30 s, and 72 °C for 45 s; followed by a final extension at 72 °C for 5 min. The PCR products were purified and subjected to single-strand sequencing using the BigDye Terminator v3.1 Cycle Sequencing Kit (Life Technologies, Waltham, MA, USA) on an ABI 3500 Genetic Analyzer (Applied Biosystems, Vancouver, BC, USA). Sequence chromatograms were manually checked and edited in BioEdit v7.2.6 [40], and SNP genotyping was subsequently performed using the same software.

### 2.8. Linkage Disequilibrium (LD) and Sum-of-Risk Score (SoR)

Linkage disequilibrium (LD) between SNPs was estimated using the *snpStats* package [41] in R [42], and a heatmap was visualized using the *corrplot* package. If r^2^ exceeded 0.80, one SNP from the pair was retained for further analysis.

Since no prior information (i.e., beta effect) was available for the genotyped SNPs in the population, their effects were assumed to contribute equally to the studied phenotype. Consequently, the sum-of-risk score (SoR) was calculated as the total count of risk alleles present in each fish [43]. The alternative allele was considered the risk allele. This analysis was performed in R.

### 2.9. Evaluating the Sum-of-Risk Score (SoR) as a Predictor Fitted in an Animal Model

The following animal model was used to evaluate the effect of the SoR as a predictor in MCMC/R:

***Y* ~ *mu* + *Diet* + *SoR* + *Z u* + *e*** (1)

where the ***Y*** is the vector of the trait, the ***Diet*** is the diet/cage (2 levels), the ***SoR*** is the sum-of-risk score for the *igf1* and *ghrii* genes. ***Diet*** and ***SoR*** were fitted as fixed effects. The ***Z*** is the incidence matrix, and ***u*** is the additive genetic effect using the Pedigree Relationship Matrix (PRM) and it is illustrated as ~*N*(0, *Pσ_α_*^2^), where the *P* is the PRM and *σ_a_*^2^ is the polygenic additive variance arising from the PRM and the ***e*** is the residual.

### 2.10. Evaluating Each SNP as a Predictor in an Animal Model

The following animal model was used to evaluate the effect of each SNP as a predictor in MCMC/R [44]:

***Y* ~ *mu* + *Diet* + *SNP* + *Z u* + *e*** (2)

where the ***Y*** is the vector of the trait, the ***Diet*** is the diet/cage (2 levels), and the ***SNP*** represents the genotype at a given SNP (15 SNPs were tested individually). ***Diet*** and ***SNP*** were fitted as fixed effects in the animal model. ***Z*** is the incidence matrix, ***u*** is the additive genetic effect using the PRM, and it is illustrated as ~*N*(0, *Pσ_α_*^2^) where the *P* is the PRM and *σ_a_*^2^ is the polygenic additive variance arising from the PRM, and ***e*** is the residual. Then, a Bonferroni correction was used in order to avoid false positive results (a = 0.05 and a = 0.1, divided by the total number of SNPs).

## 3. Results

### 3.1. Genetic Parameters and Genotype by Diet Interaction (G × D)

Descriptive statistics for the metabolic traits are illustrated in Table 5. A total of 303 fish were fed on the FM and 281 were fed on the PP. The metabolic traits, Prot_D15, Prot_D30, Chol_D15, Chol_D30, Trigl_D15, and Trigl_D30, were measured in 584 fish, and they were used to estimate the genetic parameters and to investigate the genotype by diet interaction (G × D). Heritability estimates for the metabolic traits ranged from low to moderate (Table 6). Statistically significant heritability estimates were observed for Prot_D30, Chol_D15, Trigl_D30, as well as for growth-related traits (weight and muscle fat content). Significantly positive estimates of genetic correlations were found between Trigl_D30 and both final weight (WF) and muscle fat content. Notably, genetic correlations were not statistically significant between the following trait pairs: Prot_D15 and Prot_D30, Chol_D15 and Chol_D30, Trigl_D15 and Trigl_D30. The additive genetic variance and residual variance are presented in Tables S2 and S3, respectively.

In terms of genotype-by-diet interactions, no evidence of G × D effects were detected between diets for these traits, as most of the genetic correlations were not significant (Table 7). However, six out of the eight genetic correlations between the metabolic traits under the different diets were estimated to be negative, although they were not statistically significant. Only the genetic correlation for blood Prot_D30 levels under the different diets was very high, followed by a moderate estimate for cholesterol at 15 days after the change in diet. Therefore, it would be more likely that G × D effects could be expected for most metabolic factors.

### 3.2. Genotyping and SNP Analysis of ghrii and igf1

A total of 791 polymorphisms were identified in the two genes of interest, with the majority located in intronic regions. Functional annotation of these SNPs was performed using the SnpEff tool [45] and is summarized in Table 8. All SNPs located in the 3′ UTR of both genes were selected for genotyping. Of the seventeen (17) SNPs genotyped, three (3) were found to be monomorphic and were therefore excluded from the analysis (*igf1*_ SNP1, *igf1*_ SNP12 and *ghrii*_ SNP1).

### 3.3. Family-Specific Transcriptomic Responses to a Plant-Rich Diet (PP)

The 10 selected families used to investigate transcriptomic responses were F03, F05, F06, F08, F11, F13, F14, F15, F17, and F20. The first four families (F03, F05, F06, and F08) were characterized by high weight gain and low within-family variation, whereas the remaining six families (F11, F13, F15, F17, and F20) showed low weight gain and high within-family variation. Based on Specific Growth Rate (SGR) patterns, families were further classified into three groups: Pattern A included F08, F14, and F20; Pattern B included F05, F11, F13, and F17; and Pattern C included F03, F06, and F15.

**Figure 1:**
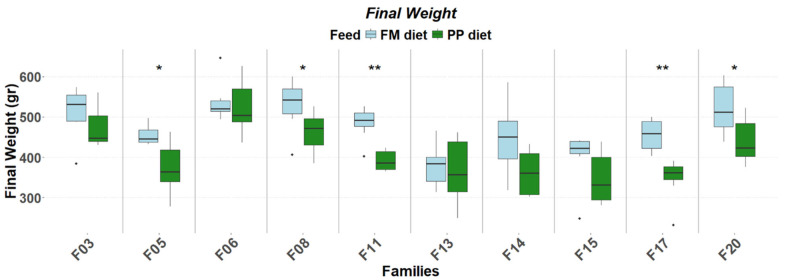
Final weights in ten selected families of extreme phenotypes. Significant changes between feeds (light blue: FM, green: PP) are marked with asterisks (*). Significance levels are presented on the plot (* < 0.05, ** < 0.01). Dots represent outliers (·).

**Figure 2:**
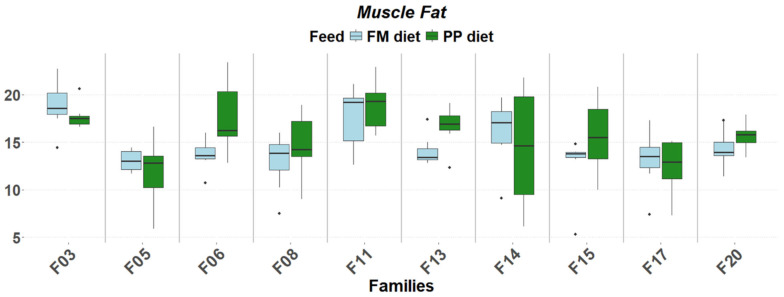
Final muscle fat contents (% body weight) in ten selected families of extreme phenotypes. Significant changes between feeds (light blue: FM, green: PP) are marked with asterisks (*). Significance levels are presented on the plot (* < 0.05). Dots represent outliers (·).

**Figure 3:**
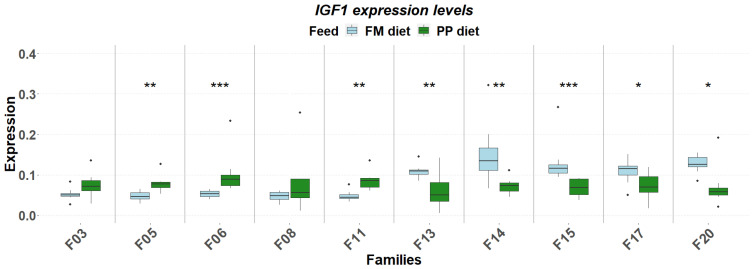
Hepatic expressions of *igf1* in ten selected families of extreme phenotypes. Significant changes between feeds (light blue: FM, green: PP) are marked with asterisks (*). Significance levels are presented on the plot (* < 0.05, ** < 0.01, *** < 0.001). Dots represent outliers (·).

**Figure 4:**
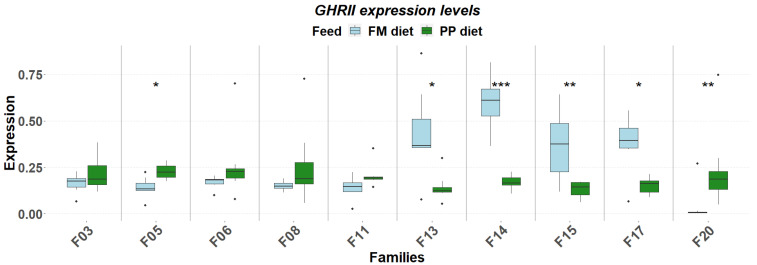
Hepatic expression of *ghrii* in ten selected families of extreme phenotypes. Significant changes between feeds (light blue: FM, green: PP) are marked with asterisks (*). Significance levels are presented on the plot (* < 0.05, ** < 0.01, *** < 0.001). Dots represent outliers (·).

**Figure 5:**
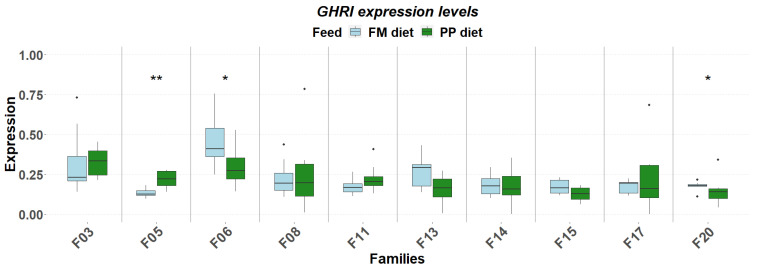
Hepatic expression of *ghri* in ten selected families of extreme phenotypes. Significant changes between feeds (light blue: FM, green: PP) are marked with asterisks (*). Significance levels are presented on the plot (* < 0.05, ** < 0.01). Dots represent outliers (·).

**Figure 6:**
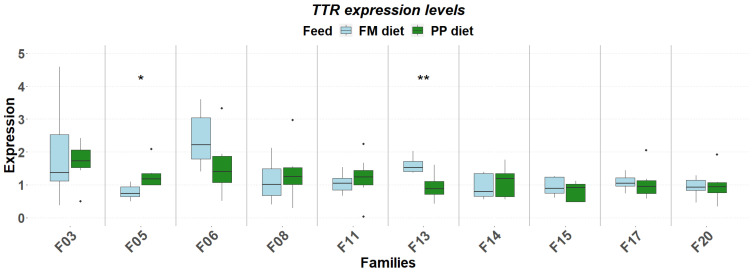
Hepatic expression of *ttr* in ten selected families of extreme phenotypes. Significant changes between feeds (light blue: FM, green: PP) are marked with asterisks (*). Significance levels are presented on the plot (* < 0.05, ** < 0.01). Dots represent outliers (·).

The detailed expression patterns revealed a significant family-specific response of these genes to the PP. The final weight supported by each feed was significantly affected by the genetic background (Figure 1). FM feed resulted in significantly higher final weight in 5 of the 10 families. Although no significant differences were detected in muscle fat content, it varied as a function of diet and genetic background, with the PP feed supporting higher fat deposition in specific families (Figure 2). Similarly, gene expression levels significantly differentiated between diets according to genetic background. A reverse response pattern to PP was observed for hepatic *igf1* and *ghrii* between families with high weight gain and low within-family variability (F03, F05, F06, F08) and families with low weight gain and high within-family variability (F11, F13, F14, F15, F17, F20), indicative of the effect of selection on the central growth regulation (Figure 3 and Figure 4). No such trend was observed in the expression of *ghri* and *ttr* (Figure 5 and Figure 6).

Significant overall and diet-specific correlations were observed between phenotypic traits and hepatic gene expression levels (Table 9). Expression levels of *igfi* and *ghrii* were negatively correlated with the final weight of fish fed on the FM. In contrast, significantly positive correlations were observed between final weight and the expression levels of *ghri*, *ghrii*, and *ttr*, with the strongest correlations detected in fish fed the PP (Table 9).

In fish fed the FM, hepatic *igf1* and *ghrii* expressions were negatively correlated with final weight. In contrast, under the PP, positive correlations were observed between final weight and the expression of *ghri*, *ghrii*, and *ttr*. These contrasting patterns suggest that hepatic endocrine gene expression does not act as a simple direct proxy for growth but rather reflects diet-dependent regulatory responses. In the nutritionally favorable FM condition, higher growth may be achieved without elevated endocrine activation, whereas under the more challenging PP, increased *ghri*/*ghrii* expression may represent a compensatory mechanism to support growth.

### 3.4. Linkage Disequilibrium (LD)

**Figure 7:**
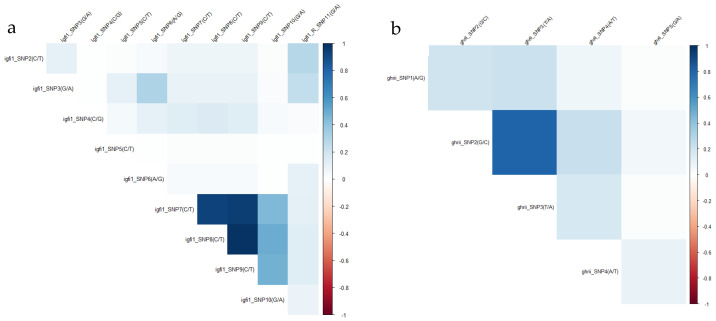
Linkage disequilibrium (r^2^) of SNPs within (**a**) *igfi* and (**b**) *ghrii* genes.

Focusing on the Linkage disequilibrium (LD) between the SNPs, two SNPs in the *ghrii* gene were found to have r^2^ = 0.81 and three SNPs in the *igf1* gene exhibited r^2^ ranging from 0.94 to 0.98 (Figure 7). These SNPs were in strong LD, and to avoid redundancy, only one SNP per LD block was selected for inclusion in the SoR (*igfi*_SNP7, *igfi*_SNP9 and *ghii*_SNP3 were excluded from the analysis).

### 3.5. Evaluating the Sum-of-Risk Score as a Predictor in an Animal Model

A significant relationship between final weight and diet (*p*-value < 0.001) was identified, while no association was detected between final weight and sum-of-risk allele score (SoR) for *igf1* and *ghrii* (*p*-value = 0.286, Table 10). Fish fed on the PP showed a 61.22-unit lower weight compared with those fed on the FM. Regarding muscle fat content, both the sum-of-risk allele score (SoR) and the diet were significantly associated with fat levels (*p*-value equal to 0.01 and 0.022, respectively, Table 10). Fish fed on the PP showed an increase of 1.09 units in fat levels, while each one-unit increase in SoR was associated with a 0.30 unit decrease in fat levels.

The SoR exhibited a statistically significant association with *ttr* expression levels (*p*-value = 0.044, Table 10). A one-unit increase in the sum-of-risk allele score (SoR) was associated with a 0.05 unit decrease in *ttr* expression levels. For all other phenotypes, the SoR did not show a significant association. On the other hand, a significant relationship between the diet and the expression levels of the metabolic traits Prot_30, Trigl_D15, Trigl_D30 and *ghrii* was identified. The consumption of the PP led to increased levels in those traits (0.02 units for Trigl_D15, 0.1 units for Trigl_D30, 0.19 units for Prot_D30, and 0.26 units for *ghrii* expression levels).

### 3.6. Evaluating the Effect on Each SNP as a Predictor in an Animal Model

In the 160 population, fish fed the FM had an average final weight of 471.32 g (SD = 73.99) and an average muscle fat content of 14.63% (SD = 3.12), and fish fed the PP had an average final weight of 407.59 g (SD = 81.43) and an average muscle fat content of 15.57% (SD = 3.87).

Table 11 presents the results of the regression analysis (model 4), including only SNPs that had *p*-values below 0.1 after Bonferroni correction. One SNP in the *igf1* gene was associated with Trigl_D15 levels. Two SNPs in the *igf1* gene were associated with *ghrii* expression levels while one SNP in *ghrii* was associated with *ttr* expression levels. The *p*-values for all the regression models are illustrated in Table S1.

To elaborate, for Trigl_D15, fish with the C/T genotype of *igf1*_SNP2 showed a 0.03-unit increase compared to those with the C/C and T/T genotypes. *igf1*_SNP2 (C/T) and *igf1*_SNP3 (G/A) showed statistically significant associations with *ghrii* expression levels. Fish with the C/T genotype of *igf1*_SNP2 showed a 0.33 unit increase in *ghrii* expression levels compared to fish with the C/C and T/T genotypes. Fish with the G/A genotype of *igf1*_SNP2 showed a 0.33 unit decrease in *ghrii* expression levels compared to those with the G/G and A/A genotypes. Fish with genotype T/T of the *ghrii*_SNP4 had a decreased effect of 0.39 units compared to fish with the C/C and T/T genotypes.

## 4. Discussion

Our study addressed a fundamental challenge in aquaculture: the identification of early, minimally invasive biomarkers that are genetically informative and predictive of growth-related performance in gilthead seabream (*S*. *aurata*). The experimental fish were fed on two diets of different ingredients designed to be isoproteinic and isolipidic as described in detail in previous work [17,20,21]. Traditionally, traits such as final weight and/or fat content are only measurable at harvest, limiting their utility in real-time decision-making and early selection schemes. By combining classical animal models with gene expression analysis and regulatory SNP mapping, this study presents an integrative biological framework for exploring how variation in key metabolic genes, such as *igf1* and *ghrii*, relates to metabolic and growth-associated traits. These findings improve our understanding of the links between early systemic metabolic status and later phenotypic outcomes, and provide a basis for future evaluation of candidate indicators in breeding-oriented aquaculture research.

Heritability reflects the proportion of phenotypic variance explained by additive genetic variance [46]. In our study, moderate heritabilities were estimated for the key blood metabolic indicators (plasma protein, cholesterol and triglycerides levels) in gilthead seabream; notably, each indicator became transiently informative at different times following dietary change (cholesterol at D15, plasma protein and triglycerides at D30), indicative of an orchestrated sequence of events triggered by the diet shift. Although these metabolic markers are influenced by environmental factors, i.e., dietary stress from plant protein (PP) diets [21], the present findings indicate that their variation is partly explained by genetic variation as well. To date, genetic contributions to blood metabolic traits have been rarely investigated in fish, with only limited evidence reported for lipid-related traits, such as n-3 PUFA composition in Atlantic salmon [47]. Collectively, the moderate heritability estimates and the influence of multiple environmental factors on these traits are consistent with a complex nature for these traits [46].

From a physiological perspective, plasma protein levels reflect amino acid availability, liver synthetic capacity, and the anabolic effects of the GH–IGF endocrine axis [27]. Moderate heritability of Prot_D30 suggests genetic variability contributes to individual variation in the ability to maintain protein homeostasis under alternative diets. Likewise, cholesterol, a critical precursor for steroid hormones and bile acids, may reflect variation in hepatic metabolism, intestinal absorption, and endocrine feedback, particularly involving thyroid hormone and corticosteroid pathways [48]. The high genetic correlation between Trigl_D30 and final body weight indicates that these traits may share part of their underlying additive genetic effects, potentially reflecting pleiotropic effects or closely linked loci. The relatively low phenotypic correlation suggests that environmental or residual factors may obscure this shared genetic signal at the observable level. Triglycerides reflect lipid turnover and storage potential. Considering that another strong genetic correlation observed was between final weight and muscle fat content, the genetic correlation between the final weight and the Trigl_30 may point to a metabolic balance favoring somatic growth through energy storage (fat) and via enhanced lipid absorption [49] and/or hepatic lipogenesis [50].

No evidence of G × D effect for these traits was detected, based on the non-significant genetic correlations. However, non-significant genetic correlations do not necessarily indicate the absence of a G × D interaction. Most of the estimated genetic correlations suggest the presence of G × D, as many deviated substantially from unity and several were negative. Nevertheless, the lack of statistical significance does not allow any inferences. Notably, certain traits such as Trigl_D30 showed different heritability estimates between diets (h^2^ = 0.79 PP vs. 0.50 FM). These differences, along with the general G × D outcome, could have been influenced by the limited sample size after dividing the fish into diet groups. In a previous study, two genotypes of gilthead sea bream, one of which selected for faster growth during winter, were fed on either a FM or a plant meal-based diet, to observe no main dietary effects on growth rates or condition factor; however, the selected strain exhibited differentiated intestinal morphology and increased digestive capacity when fed on the plant-based diet [51]. In another study, gilthead seabream selected for growth differentiated in terms of gut microbiota composition and gut transcriptomics, and this differentiation was influenced by diet [52]. In a contrasting study, gilthead sea bream selected for fast growth exhibited higher pepsin and chymotrypsin activities than the reference strain, yet these activities were not influenced by the diet [18]. It becomes evident that G × D interactions may reveal on specific physiological indicators, whereas others are more affirmatively driven by the genotype.

When considering all the results generated in this study, the regulation of metabolic traits appears to reflect a complex interplay between genetic factors (i.e., substantial and significant heritability estimates and SNP effects within the 3′ UTRs) and environmental influences (diet effects on growth performance and transcriptional profiles). To this end, the present findings add support to orienting selective breeding towards optimizing metabolic efficiency along with optimizing growth. Traits that are related to metabolic homeostasis, such as stable hepatic function, could serve as novel selection objectives to increase fish meal substitution with emerging feed ingredients in gilthead seabream [18,21]. As breeding programs advance, the ability of selected fish for fast growth to utilize alternative feed substrates has gained attention for promoting economic and environmental sustainability [53].

The Sum-or-risk score (SoR) and the diet effect were used to investigate the effect of the genetic background on the expression of selected hepatic genes (transcriptomic data). Our results show that a higher SoR score, reflecting an increased number of risk alleles in the 3′ untranslated regions (UTRs) of *igf1* and *ghrii*, was significantly associated with a reduction in muscle fat and decreased hepatic expression of *ttr* (Table 10). Since the 3′ UTRs are key regulatory regions that influence transcript stability, localization, and translational efficiency, it is plausible that these variants may interfere with microRNA binding sites or RNA-binding protein recognition motifs [54,55].

In our study, all SNPs were assigned equal weights for estimation of the SoR score. This approach was considered more appropriate because the newly identified SNPs in the two 3′ UTRs are not included in the 30K MedFISH SNP array [56], and no beta effect estimates from a GWAS analysis [57] were available for these variants. Using equal weights therefore represented a safer option, as estimating SNP effects from the same population would likely introduce bias into the SoR score. Consequently, the observed effects of these SNPs on the expression of selected hepatic genes could not be directly applicable into selection programs. Nevertheless, their functional relevance could be further investigated through in vitro assays or in silico motif prediction analyses to assess potential interference with microRNA binding sites or RNA-binding proteins.

The observed negative correlation between SoR score and muscle fat suggests that accumulation of specific allelic variants may impair lipid deposition pathways, possibly through dysregulation of the GH-IGF axis. *igf1* is a principal effector of anabolic activity, promoting nutrient assimilation, lipogenesis, and protein synthesis, while *ghrii* modulates the sensitivity of target tissues to circulating GH [58]. The reduction in *ttr* expression in individuals with high SoR score may further exacerbate metabolic inefficiency, given the role of transthyretin in transporting thyroid hormones, which are essential regulators of basal metabolic rate, mitochondrial activity, and nutrient partitioning [59,60,61]. A study set to elucidate the role of GHR in systemic insulin resistance used hepatic samples from obese humans, mouse strains and primary mouse hepatocytes to produce evidence that hepatic GHR overexpression promoted lipolysis in white adipose tissue and repressed glucose utilization in skeletal muscle through increased circulating levels of RBP4. The study revealed that hepatic GHR acted to increase the transcription of both *rbp4* and *ttr* through different pathways. Moreover, obesity and consumption of a high-fat diet pushed the transcription of *ghr* to higher levels and higher levels of GHR were associated with higher fatty acid oxidation [62]. A significantly positive correlation between the transcription levels of *ghri*, *ghrii* and *ttr* was also revealed in the present study (Table 9), indicating that a similar mechanism may exist in gilthead sea bream.

The correlations were stronger in the PP, as it was also evident the link between *ghrii* expression (↑0.26), elevated circulating triglyceride levels at D30 (↑0.10) and increased muscle fat (↑1.09), indicating that the plant-rich feed triggered an endocrine and metabolic reprogramming. These shifts suggest an effort to maintain growth under suboptimal nutrition, though its effectiveness likely varies according to the genetic background of the individual fish. Indeed, the transcriptomic response to the PP was not uniform across different families. Families previously selected for high growth and low within-family variability showed a downregulation of both *igf1* and *ghrii* under the PP, whereas low-performing families exhibited upregulation of these same genes (Figure 3 and Figure 4). These observations point to a G × D interaction at the transcriptomic level, where prior genetic selection has influenced endocrine feedback sensitivity and transcriptional adaptability in accordance with previous findings [19,20,25].

The analysis of individual SNPs located in the 3′ UTRs of *igf1* and *ghrii* revealed locus-specific associations with both gene expression and metabolic traits, highlighting their likely role as functional regulators of endocrine responses. These variants are well-positioned to exert cis-regulatory control by influencing mRNA stability, microRNA binding, or translational efficiency, post-transcriptional mechanisms that can finely tune gene expression in response to internal and external stimuli [55,63,64,65,66,67]. One such variant, IGF1_ SNP2 (C/T), was associated with both increased hepatic *ghrii* expression and elevated circulating triglyceride levels at D15. This suggests that allelic variation at this regulatory site may modulate *igf1* transcript dynamics, altering downstream sensitivity of the GH–IGF system. The link between *igf1* and *ghrii* expression likely reflects a feedback mechanism within the endocrine axis, where IGF1 availability influences growth hormone receptor regulation, as observed in other vertebrates [67,68]. Likewise, *ghrii*_ SNP4 (A/T) was significantly associated with lower hepatic expression of *ttr*, providing additional indications of a mechanistic regulation of *ttr* expression by *ghrii* with implications for thyroid hormone distribution and energy metabolism. Importantly, these SNP-level effects are consistent with broader polygenic trends. A cumulative polygenic risk score based on 3′ UTR variants in *igf1* and *ghrii* was negatively associated with both fat content and *ttr* expression, suggesting that these regulatory alleles may contribute to a less efficient metabolic phenotype.

## 5. Conclusions

In conclusion, this study demonstrates that early blood metabolic indicators and liver expression of GH–IGF axis genes harbor heritable variation and diet-dependent plasticity in a commercial gilthead seabream breeding population. The integration of quantitative genetics with candidate gene expression and 3′ UTR regulatory variants provides a biological framework for exploring possible associations between minimally invasive biomarkers and long-term growth-related traits under alternative diets. Future work should combine genome-wide genotyping efforts, broader endocrine and metabolic profiling (e.g., circulating GH, IGF1, thyroid hormones, liver lipidomics), and multi-tissue transcriptomics to identify robust marker panels and breeding indices that explicitly incorporate metabolic homeostasis and feed efficiency under low fish meal diets. Such integrative approaches will be essential for designing selective breeding programs that support both productivity and sustainability in Mediterranean aquaculture.

## Figures and Tables

**Table 1:** Studied phenotypes.

Phenotype	Description
WF (g)	Final weight at 549 DPH
FAT (% body weight)	Muscle fat content at 549 DPH
Prot_D15 (mg/mL)	Serum protein content 15 days after dietary shift (240 DPH)
Prot_D30 (mg/mL)	Serum protein content, 30 days after dietary shift (255 DPH)
Cholesterol_D15 (mg/mL)	Serum cholesterol levels, 15 days after dietary shift (240 DPH)
Cholesterol_D30 (mg/mL)	Serum cholesterol levels 30 days after dietary shift (255 DPH)
Triglycerides_D15 (mg/mL)	Serum triglyceride levels 15 days after dietary shift (240 DPH)
Triglycerides_D30 (mg/mL)	Serum triglyceride levels 30 days after dietary shift (255 DPH)

**Table 2:** Genes selected for differential expression analysis through Real Time PCR.

Gene ID	Gene Name	Gene Description	Forward Primer	Reverse Primer	Product Size (bp)
ENSSAUG00010003114	*rpl13a*	Ribosomal protein L13a	TCTGGAGGACTGTCAGGGGCATGC	AGACGCACAATCTTAAGAGCAG	148
ENSSAUG00010000811	*rps18*	40S ribosomal protein S18	AGGGTGTTGGCAGACGTTAC	GAGGACCTGGCTGTATTTGC	197
ENSSAUG00010018560	*ef1a*	Elongation factor 1-alpha, somatic form	TCAAGGGATGGAAGGTTGAG	AGTTCCAATACCGCCGAT	152
ENSSAUG00010015109	*igf1*	*Insulin growth factor 1*	CGAGCCCAGAGACCCTTGT	AGTCTTGGCAGGTGCACAGTA	155
ENSSAUG00010018083	*ghrii*	*Growth Hormone Receptor II*	GACAAGCTCACAGACCTGGAC	TTGATTTGGGATGAGAGGATG	174
ENSSAUG00010007903	*ttr*	*Transthyretin*	CCAGCAGGAGTGTATCGTGT	TGGTGGTGTAGGAGAACGGA	163
ENSSAUG00010008479	*ghri*	*Growth Hormone receptor I*	TTGGGCATCCTCATACTCATC	TGGTAGAAATCTGGC	203

**Table 3:** SNPs position for each gene.

Gene Name	Ensembl ID	Chromosome	Genomic Coordinates	SNP	Position	Reference Allele	Alternative Allele	**Ensembl ID**
*ghrii*	ENSSAUG00010018083	12		*ghrii*_F_ SNP1	5,883,250	A	G	ENSSAUG00010018083
				*ghrii*_F_ SNP2	5,883,262	G	C	ENSSAUG00010018083
			*5,862,586–5,883,486*	*ghrii*_F_ SNP3	5,883,269	T	A	ENSSAUG00010018083
				*ghrii*_F_ SNP4	5,883,326	A	T	ENSSAUG00010018083
				*ghrii*_F_ SNP5	5,883,374	G	A	ENSSAUG00010018083
*igf1*	ENSSAUG00010015109	14	*20,269,968–20,287,774*	*igf1*_R_ SNP1	20,287,176	T	C	ENSSAUG00010015109
				*igf1*_R_ SNP2	20,287,468	C	T	ENSSAUG00010015109
				*igf1*_R_ SNP3	20,287,523	G	A	ENSSAUG00010015109
				*igf1*_R_ SNP4	20,287,547	C	G	ENSSAUG00010015109
				*igf1*_R_ SNP5	20,287,586	C	T	ENSSAUG00010015109
				*igf1*_R_ SNP6	20,287,590	A	G	ENSSAUG00010015109
				*igf1*_R_ SNP7	20,287,637	C	T	ENSSAUG00010015109
				*igf1*_R_ SNP8	20,287,644	C	T	ENSSAUG00010015109
				*igf1*_R_ SNP9	20,287,649	C	T	ENSSAUG00010015109
				*igf1*_R_ SNP10	20,287,662	G	A	ENSSAUG00010015109
				*igf1*_R_ SNP11	20,287,697	G	A	ENSSAUG00010015109
				*igf1*_R_ SNP12	20,287,724	C	T	ENSSAUG00010015109

**Table 4:** Primers designed and used in genotyping.

Gene ID	Gene Name	Gene Description	Primer Name	Forward Primer	Reverse Primer
ENSSAUG00010015109	*igf1*	*Insulin growth factor 1*	IGF1F/IGF1R	ACAGAGAATCAAATTAACCAGAAGC	ATGTGTGTTTGTGCGCTGTT
ENSSAUG00010018083	*ghrii*	*Growth Horomone Receptor II*	GHRII_F/GHRII_R	CGAAGACCATGCCAACACCAG	TCGATGTTACGGCCCTGTCT

**Table 5:** Descriptive statistics for the metabolic traits and growth per diet.

Diet	FM							
Trait	WF (g)	Prot_D15 (mg/mL)	Prot_D30 (mg/mL)	Chol_D15 (mg/mL)	Chol_D30 (mg/mL)	Trigl_D15 (mg/mL)	Trigl_D30 (mg/mL)	Muscle Fat (%)
Number of measurements	303	303	303	303	303	303	303	206
Mean	474.39	35.63	29.76	36.15	38.09	47.98	51.03	15.29
Sd	87.18	14.31	11.51	9.70	9.44	10.14	11.34	4.64
Min	197.00	5.01	10.20	20.05	20.05	30.84	30.87	3.00
Max	717.00	76.51	74.87	94.19	93.96	105.76	106.21	28.30
**Diet**	**PP**							
Number of measurements	279	281	281	281	281	281	281	193
Mean	393.87	35.24	54.86	37.51	37.93	51.90	63.35	14.71
Sd	93.41	13.17	19.19	10.42	9.02	12.16	20.72	5.23
Min	138.00	7.87	5.65	22.08	21.18	32.89	32.21	1.90
Max	684.00	81.42	114.35	106.35	68.95	145.04	167.20	26.80

**Table 6:** Genetic parameters for the metabolic factors; heritability is on the diagonal in bold; genetic (in green) and phenotypic (in blue) correlations are above and below the diagonal, respectively. Standard errors are illustrated in parentheses.

	WF	Prot_D15	Prot_D30	Chol_D15	Chol_D30	Trigl_D15	Trigl_D30	MUSCLE FAT
WF	0.55 (0.16) *	−0.63 (0.57)	−0.22 (0.36)	0.04 (0.39)	−0.12 (0.48)	−0.11 (0.65)	0.69 (0.26) *	0.63 (0.31) *
Prot_D15	−0.15 (0.05)	**0.11 (0.06)**	−0.20 (0.61)	0.14 (0.54)	0.41 (0.65)	−0.62 (1.02)	−0.12 (0.51)	−0.78 (0.9)
Prot_D30	−0.08 (0.06)	0.11 (0.05)	0.30 (0.11) *	0.45 (0.50)	0.53 (0.48)	0.42 (0.80)	0.04 (0.39)	−0.35 (0.42)
Chol_D15	0.03 (0.06)	0.13 (0.05)	0.07 (0.05)	0.23 (0.09) *	0.54 (0.50)	0.39 (0.77)	0.30 (0.39)	0.02 (0.46)
Chol_D30	−0.03 (0.05)	−0.03 (0.04)	0.04 (0.05)	0.16 (0.04)	**0.11 (0.06)**	−0.38 (1.07)	0.69 (0.72)	−0.33 (0.89)
Trigl_D15	−0.02 (0.05)	−0.04 (0.04)	−0.05 (0.05)	0.04 (0.04)	0.00 (0.04)	**0.06 (0.04)**	−0.31 (1.07)	0.31 (1.16)
Trigl_D30	0.17 (0.07)	0.01 (0.05)	−0.02 (0.06)	0.08 (0.05)	0.13 (0.05)	0.02 (0.05)	0.32 (0.11) *	0.19 (0.67)
MUSCLE FAT	0.59 (0.05)	−0.11 (0.06)	−0.08 (0.06)	−0.01 (0.06)	−0.05 (0.06)	−0.01 (0.06)	−0.01 (0.06)	0.32 (0.13) *

* Statistically significant estimates.

**Table 7:** Genotype by Diet interaction for the metabolic factors.

Trait	Heritability PP	Heritability FM	Genetic Correlation
Prot_D15	0.48 (0.16) *	0.29 (0.12) *	−0.36 (0.47)
Prot_D30	0.77 (0.20) *	0.01 (0.01)	1.00 (0.18)
Chol_D15	0.23 (0.11) *	0.31 (0.13) *	0.69 (0.52)
Chol_D30	0.34 (0.14) *	0.18 (0.10)	−0.06 (0.66)
Trigl_D15	0.11 (0.08)	0.26 (0.12) *	−0.18 (0.85)
Trigl_D30	0.79 (0.20) *	0.50 (0.16) *	−0.06 (0.40)

* Statistically significant estimates.

**Table 8:** Total number of SNPs per category for each gene.

Gene	5′ UTR Variants	Intron Variants	Missense Variants	Synonymous Variants	3′ UTR Variants
*igf1*	-	425	3	1	12
*ghrii*	5	319	8	11	5

**Table 9:** Correlations between growth performance traits and endocrine gene expression levels under different dietary treatments. Correlations are presented for all fish combined (Corr), as well as separately for fish fed the fish meal-based diet (FM) and the plant-protein diet (PP). Asterisks denote statistical significance (*p* < 0.05 *, *p* < 0.01 **, *p* < 0.001 ***, *p* < 0.0001 ****).

	*ttr*	Muscle Fat	*igf1*	*ghrii*	*ghri*	
Corr	0.044	0.230 **	−0.200 *	−0.068	0.157	WF
FM	0.002	0.228 *	−0.311 **	−0.351 **	0.122	
PP	0.106	0.398 ***	−0.072	0.218	0.217	
Corr		0.100	0.302 ***	0.347 ***	0.769 ***	*ttr*
FM		0.110	0.017	0.112	0.803 ***	
PP		0.094	0.677 ****	0.661 ***	0.729 ***	
Corr			−0.092	0.045	0.124	Muscle Fat
FM			−0.140	0.093	0.087	
PP			0	0.033	0.0177	
Corr				0.520 ***	0.260 **	*igf1*
FM				0.427 ***	0.062	
PP				0.615 ***	0.507 ***	
Corr					0.283 ***	*ghrii*
FM					0.083	
PP					0.554 ***	

**Table 10:** Regression coefficients (β), 95% confidence intervals, and significance levels from linear regression model 3 examining the effects of diet and SoR on the studied phenotypes.

**Trait/Dependent variable**	**WF**		**MUSCLE FAT**		**Prot_D15**		**Prot_D30**	
	Regression coefficient (β) (95% CI)	*p*-value	Regression coefficient (β) (95% CI)	*p*-value	Regression coefficient (β) (95% CI)	*p*-value	Regression coefficient (β) (95% CI)	*p*-value
(Intercept)	486.17 (426.89,536.02)	<0.001	17.35 (14.82,20.18)	<0.001	1.48 (1.35,1.59)	<0.001	1.46 (1.33,1.59)	<0.001
PP	−61.22 (−78.98,−40.88)	<0.001	1.09 (0.21,2.03)	0.022	0.01 (−0.06,0.05)	0.806	0.19 (0.13,0.25)	<0.001
SoR	−2.73 (−7.70,2.28)	0.286	−0.39 (−0.64,−0.11)	0.01	0.00 (−0.01,0.02)	0.612	0.00 (−0.02,0.01)	0.878
**Trait/Dependent variable**	**Chol_D15**		**Chol_D30**		**Trigl_D15**		**Trigl_D30**	
	Regression coefficient (β) (95% CI)	*p*-value	Regression coefficient (β) (95% CI)	*p*-value	Regression coefficient (β) (95% CI)	*p*-value	Regression coefficient (β) (95% CI)	*p*-value
(Intercept)	1.51 (1.45,1.57)	<0.001	1.57 (1.50,1.64)	<0.001	1.66 (1.62,1.71)	<0.001	1.71 (1.63,1.80)	<0.001
PP	−0.01 (−0.04,0.02)	0.492	0.02 (0.00,0.05)	0.1	0.02 (0.00,0.04)	0.046	0.10 (0.06,0.13)	<0.001
SoR	0.01 (0.00,0.01)	0.114	0.00 (−0.01,0.01)	0.52	0.00 (0.00,0.01)	0.718	0.00 (−0.01,0.01)	0.57
**Trait/Dependent variable**	***igf1***		***ghrii***		***ghri***		***ttr***	
	Regression coefficient (β) (95% CI)	*p*-value	Regression coefficient (β) (95% CI)	*p*-value	Regression coefficient (β) (95% CI)	*p*-value	Regression coefficient (β) (95% CI)	*p*-value
(Intercept)	−2.29 (−2.60,−1.97)	<0.001	−1.59 (−2.26,−1.00)	<0.001	−1.14 (−1.62,−0.69)	<0.001	0.36 (0.04,0.70)	0.036
PP	−0.11 (−0.25,0.03)	0.148	0.26 (0.06,0.51)	0.026	−0.20 (−0.41,0.03)	0.086	−0.04 (−0.18,0.11)	0.604
SoR	0.00 (−0.04,0.04)	0.954	−0.03 (−0.10,0.03)	0.324	−0.04 (−0.10,0.02)	0.216	−0.05 (−0.09,−0.01)	0.044

**Table 11:** Regression coefficients (β), 95% confidence intervals, and significance levels from linear regression model 4 examining the effects of diet and SNPs on the studied phenotypes.

Trait/Dependent Variable	Predictors in the Model	Regression Coefficient (β)	l-95%CL	u-95%CL	*p*-Value
Trigl_D15	(Intercept)	1.66	1.63	1.68	<0.001
	PP	0.02	0.00	0.04	0.068
	*igf1*_ SNP2(CT)	0.03	0.01	0.05	0.006 *
	*igf1*_ SNP2(TT)	0.05	−0.02	0.12	0.164
*ghrii* expression levels	(Intercept)	−1.63	−2.01	−1.17	<0.001
	PP	0.29	0.06	0.52	0.01
	*igf1*_ SNP3(GA)	−0.33	−0.54	−0.08	0.004 *
	*igf1*_ SNP3(GG)	−0.45	−0.79	−0.07	0.01
	(Intercept)	−1.94	−2.38	−1.51	<0.001
	PP	0.24	0.02	0.45	0.028
	*igf1*_ SNP2(CT)	0.33	0.11	0.54	0.001 **
	*igf1*_ SNP2(TT)	0.33	−0.38	0.92	0.326
*ttr* expression levels	(Intercept)	0.12	−0.04	0.30	0.16
	PP	−0.03	−0.17	0.11	0.69
	*ghrii*_ SNP4(AT)	−0.08	−0.26	0.07	0.32
	*ghrii*_ SNP4(TT)	−0.39	−0.62	−0.15	0.002 **

** a = 0.05/15 = 0.003, * a = 0.1/15=0.006.

## Data Availability

Whole genome and transcriptome sequencing data used in this study are available through SRA (BioProject ID PRJNA1050571 and PRJNA1064006 respectively).

## Supplementary Materials

The following supporting information can be downloaded at https://www.mdpi.com/article/10.3390/genes17050550/s1: Supplementary Material S1: Descriptive statistics of the gene expression; Table S1: *p*-values per trait per SNP, Table S2: Additive variances estimated from models 1 and 2 (Table S2), Table S3: Residual variance estimated from Models 1 and 2 (Table S3).
