# Supplementary material for: Genetic and Dietary Influences on Metabolic Traits in Gilthead Seabream (Sparus aurata)
Source: Genes (Basel). 2026 May 5;17(5):550. doi: 10.3390/genes17050550 (PMC13206124; doi:10.3390/genes17050550)
Supplement: Supplementary file 1 [file genes-17-00550-s001.zip › Supplementary Material S1.pdf]

Supplementary Material. Descriptive statistics of the gene expression

**Diet**	**FM diet**			
Trait	*igf1*	*ghrii*	*ghri*	*ttr*
Number of measurements	159	159	159	159
Mean	0.010508	0.107643	0.069048	2.187292
Sd	0.001706	0.017714	0.011781	0.377926
Min	0.00069	1.36E-05	0.009622	0.141919
Max	0.103324	0.745675	0.571894	21.07694
**Diet**	**PP diet**			
Trait	*igf1*	*ghrii*	*ghri*	*ttr*
Number of measurements	159	159	159	159
Mean	0.007575	0.05548	6.55E-02	1.760288
Sd	0.001142	0.011089	1.07E-02	0.209834
Min	2.72E-05	0.0025	2.37E-06	0.000921
Max	0.064304	0.559992	6.15E-01	11.09358
