# Supplementary material for: Genetic and Dietary Influences on Metabolic Traits in Gilthead Seabream (Sparus aurata)
Source: Genes (Basel). 2026 May 5;17(5):550. doi: 10.3390/genes17050550 (PMC13206124; doi:10.3390/genes17050550)
Supplement: Supplementary file 1 [file genes-17-00550-s001.zip › Table S1.pdf]

Table S1. *P*-values per trait per SNP

SNP	Chr	W6	FAT	Brad_D15	Brad_D30	Chol_D15	Chol_D30	Trigl_D15	Trigl_D30	*Igf1* expression levels	*ghrii* expression levels	*ghri* expression levels	*ttr* expression levels
IGFI1_SNP2.C.T.1	14	0.13	0.032	0.772	0.242	0.038	0.578	0.006	0.344	0.288	0.001	0.248	0.17
IGFI1_SNP2.C.T.2	14	0.082	0.046	0.692	0.952	0.87	0.716	0.164	0.97	0.502	0.326	0.012	0.09
IGFI1_SNP3.G.A.1	14	0.392	0.918	0.13	0.548	0.26	0.62	0.024	0.452	0.264	0.004	0.17	0.55
IGFI1_SNP3.G.A.2	14	0.138	0.454	0.3	0.166	0.038	0.776	0.896	0.564	0.18	0.01	0.622	0.348
IGFI1_SNP4.C.G.1	14	0.422	0.276	0.982	0.53	0.826	0.77	0.786	0.056	0.596	0.248	0.438	0.932
IGFI1_SNP4.C.G.2	14	0.308	0.644	0.96	0.44	0.194	0.608	0.174	0.022	0.678	0.986	0.848	0.916
IGFI1_SNP5.C.T.1	14	0.93	0.806	0.752	0.75	0.5	0.89	0.138	0.128	0.8	0.9	0.544	0.326
IGFI1_SNP5.C.T.2	14	0.93	0.662	0.2	0.202	0.962	0.966	0.61	0.858	0.61	0.942	0.76	0.938
IGFI1_SNP6.A.G.1	14	0.774	0.478	0.636	0.048	0.56	0.926	0.402	0.188	0.868	0.488	0.66	0.416
IGFI1_SNP6.A.G.2	14	0.218	0.934	0.712	0.016	0.272	1	0.49	0.088	0.27	0.506	0.4	0.45
IGFI1_SNP7.C.T.1	14	0.702	0.932	0.134	0.726	0.668	0.252	0.288	0.84	0.076	1	0.64	0.89
IGFI1_SNP7.C.T.2	14	0.502	0.486	0.224	0.774	0.076	0.696	0.194	0.404	0.64	0.93	0.546	0.672
IGFI1_SNP8.C.T.1	14	0.558	0.968	0.102	0.91	0.594	0.278	0.278	0.758	0.108	0.838	0.604	0.752
IGFI1_SNP8.C.T.2	14	0.526	0.53	0.236	0.736	0.076	0.696	0.178	0.432	0.646	0.848	0.48	0.716
IGFI1_SNP9.C.T.1	14	0.802	0.786	0.128	0.828	0.548	0.208	0.316	0.95	0.166	0.726	0.506	0.862
IGFI1_SNP9.C.T.2	14	0.55	0.51	0.248	0.804	0.05	0.754	0.248	0.42	0.69	0.848	0.566	0.744
IGFI1_SNP10.G.A.1	14	0.268	0.236	0.862	0.182	0.986	0.826	0.936	0.228	0.488	0.634	0.172	0.412
IGFI1_SNP10.G.A.2	14	0.446	0.054	0.48	0.324	0.89	0.4	0.638	0.24	0.742	0.354	0.266	0.438
IGFI1_SNP11.G.A.1	14	0.4	0.342	0.464	0.526	0.288	0.498	0.136	0.334	0.708	0.68	0.734	0.142
IGFI1_SNP11.G.A.2	14	0.288	0.074	0.646	0.342	0.306	0.4	0.23	0.216	0.71	0.126	0.012	0.232
GHRII_ SNP2.G.C.1	12	0.192	0.904	0.204	0.69	0.224	0.446	0.494	0.036	0.294	0.678	0.358	0.376
GHRII _SNP2.G.C.2	12	0.164	0.352	0.228	0.636	0.476	0.65	0.834	0.046	0.262	0.584	0.562	0.166
GHRII_SNP3.T.A.1	12	0.624	0.73	0.292	0.372	0.432	0.836	0.04	0.234	0.472	0.742	0.404	0.07
GHRII_SNP3.T.A.2	12	0.608	0.63	0.31	0.904	0.844	0.54	0.256	0.19	0.394	0.768	0.64	0.136
GHRII_SNP4.A.T.1	12	0.358	0.662	0.014	0.832	0.63	0.776	0.634	0.678	0.026	0.802	0.378	0.398

GHRII_SNP4.A.T.2	12	0.964	0.11	0.4	0.416	0.036	0.69	0.064	0.94	0.22	0.736	0.372	0.002
GHRII_SNP5.G.A.1	12	0.382	0.488	0.886	0.722	0.77	0.956	0.64	0.632	0.246	0.974	0.746	0.706
GHRII_SNP5.G.A.2	12	0.366	0.364	0.874	0.564	0.682	0.856	0.466	0.814	0.184	0.818	0.786	0.66
