# Supplementary material for: Genetic and Dietary Influences on Metabolic Traits in Gilthead Seabream (Sparus aurata)
Source: Genes (Basel). 2026 May 5;17(5):550. doi: 10.3390/genes17050550 (PMC13206124; doi:10.3390/genes17050550)
Supplement: Supplementary file 1 [file genes-17-00550-s001.zip › Table S2.pdf]

Table S2. Additive variances estimated from models 1 and 2

Additive genetic effect	WF	Prot_D15	Prot_D30	Chol_D15	Chol_D30	Trigl_D15	Trigl_D30	FAT
WF	4804.40	-209.7	-128.79	12.931	-26.685	-20.905	437.01	128.11
Prot_D15		21.83	-7.9207	3.1693	6.0557	-8.3432	-5.3917	-10.41
Prot_D30			74.30	18.239	14.695	9.2091	3.4845	-8.46
Chol_D15				23.03	7.8704	5.125	13.241	0.26
Chol_D30					9.57	-3.4773	18.772	-3.03
Trigl_D15						7.87	-7.998	2.44
Trigl_D30							86.92	4.90
FAT								8.11
