# Supplementary material for: Genetic and Dietary Influences on Metabolic Traits in Gilthead Seabream (Sparus aurata)
Source: Genes (Basel). 2026 May 5;17(5):550. doi: 10.3390/genes17050550 (PMC13206124; doi:10.3390/genes17050550)
Supplement: Supplementary file 1 [file genes-17-00550-s001.zip › Table S3.pdf]

Table S3. Residual variance estimated from models 1 and 2

Residual	WF	Prot_D15	Prot_D30	Chol_D15	Chol_D30	Trigl_D15	Trigl_D30	FAT
WF	3885.90	18.018	17.689	12.958	3.2101	-1.1252	-181.89	145.74
Prot_D15		168.70	31.527	14.679	-10.034	1.4707	6.6814	2.99
Prot_D30			170.84	-7.9136	-9.1474	-17.417	-8.146	2.32
Chol_D15				78.55	7.2088	-0.77126	0.76143	-0.81822
Chol_D30					76.48	3.5186	0.61106	0.51528
Trigl_D15						116.85	10.974	-3.0491
Trigl_D30							183.40	-5.6778
FAT								17.45
